# The roles of HLA-DQB1 gene polymorphisms in hepatitis B virus infection

**DOI:** 10.1186/s12967-018-1716-z

**Published:** 2018-12-18

**Authors:** Guojin Ou, Haixia Xu, Hao Yu, Xiao Liu, Liu Yang, Xin Ji, Jue Wang, Zhong Liu

**Affiliations:** 1 Department of Laboratory Medicine, West China Second University Hospital, Chengdu, Sichuan China; 2 Key Laboratory of Birth Defects and Related Diseases of Women and Children (Sichuan University), Ministry of Education, Chengdu, Sichuan China; 3 Clinical Transfusion Research Center, Institute of Blood Transfusion, CAMS & PUMC, Chengdu, Sichuan China; 4 Key Laboratory of Transfusion Adverse Reactions, CAMS, Chengdu, Sichuan China; 5 Peoples Hospital of Deyang City, Dengyang, Sichuan China; 6 Tianfu New District People’s Hospital, Chengdu, Sichuan China

**Keywords:** HLA-DQB1, HBV susceptibility, Spontaneous clearance, mRNA expression

## Abstract

**Background:**

Infection with the hepatitis B virus (HBV) is an independent risk factor for liver cirrhosis and hepatocellular carcinoma, polymorphisms in HLA-DQB1 play an important role in HBV infections.

**Methods:**

This study examined the relationships between HLA-DQB1 alleles and HBV infection susceptibility among 256 HBV carriers and 433 healthy controls. Venous blood samples were subjected to DQB1 high-resolution typing and testing for interferon-gamma, interleukin-4 (IL-4), interleukin-10, and DQB1 mRNA expression. A meta-analysis was also performed using relevant case–control studies that evaluated the associations of HLA-DQB1 alleles with HBV infection and clearance.

**Results:**

We found that HLA-DQB1*06:03 protected against HBV infection. Levels of IFN-γ and IL-4 were significantly elevated in HBV cases with HLA-DQB1*06:05 (vs. HLA-DQB1*05:03), and the HBV group had higher DQB1 mRNA expression than the healthy control group with HLA-DQB1*05:03 and HLA-DQB1*06:02. The meta-analysis revealed that HLA-DQB1*04:01, HLA-DQB1*05:02, HLA-DQB1*05:03, and HLA-DQB1*06:01 were risk factors for HBV infection susceptibility, while HLA-DQB1*05:01, HLA-DQB1*06:03, and HLA-DQB1*06:04 protected against HBV infection. Spontaneous HBV clearance was associated withHLA-DQB1*06:04, while chronic HBV infection was associated with HLA-DQB1*02:01 and HLA-DQB1*05:02.

**Conclusion:**

DBQ1 typing can be used to identify patients who have elevated risks of HBV infection (i.e., patients with HLA-DQB1*04:01, HLA-DQB1*05:02, HLA-DQB1*05:03, and HLA-DQB1*06:01) or elevated risks of chronic HBV infection (i.e., patients with HLA-DQB1*02:01 and HLA-DQB1*05:02).

**Electronic supplementary material:**

The online version of this article (10.1186/s12967-018-1716-z) contains supplementary material, which is available to authorized users.

## Background

Infection with the hepatitis B virus (HBV) is an independent risk factor for liver cirrhosis and hepatocellular carcinoma [1]. There are > 68 million HBV carriers in China, and a national survey revealed that the seroprevalence rate was 7.18% in 2006 [2]. The HBV vaccine became widespread in China during 1992, and the seroprevalence of hepatitis B surface antigen (HBsAg) has decreased to approximately 5.49% in 2015 [3]. Nevertheless, given the size of the Chinese population, approximately 93 million people carry the HBsAg and approximately 30 million people have HBV-related diseases [4]. Therefore, HBV infection is associated with a significant public health burden in China [5].

Multiple factors influence the risk of chronic HBV infection or spontaneous HBV clearance, such as age, location, sex, body mass index, ethnicity, viral mutation, HBV virus genotype, host genetic variations, and host immune responses. In this context, human leukocyte antigens (HLA) play key roles in mediating the immune response. For example, HLA-I is present on the surface of all nucleated cells, where it presents exogenous antigens and is recognized by T cell receptors, which facilitates the adaptive immune response. In addition, HLA-II is expressed on the surface of antigen-presenting cells (APCs), which present endogenous antigens and are recognized by T-cell receptors as part of the adaptive immune response. Several polymorphisms in the HLA loci, especially HLA-II, are related to HBV infection susceptibility or spontaneous HBV clearance [6–10]. Furthermore, HLA-DQB1 gene polymorphisms are associated with the progression of human immunodeficiency virus infection [11], HCV infection susceptibility and spontaneous clearance [12], coronary artery disease [13], rheumatoid arthritis [14], and other diseases.

Previous studies [15] have demonstrated that the beta 1 subunit of the HLA-DQ surface receptor (HLA-DQB1) is associated with the immunological response to the hepatitis B vaccine. For example, increased antibody responses were observed in cases with HLA-DQB1*05:01 and HLA-DQB1*0602, while decreased antibody responses were observed in cases with HLA-DQB1*02. Furthermore, cases with HLA-DRB1*1301 and HLA-DQB1*0602 had high response specificity but low response sensitivity. Moreover, a previous meta-analysis [16] has indicated that some HLA-DQB1 polymorphisms (HLA-DQB1*02:01, HLA-DQB1*03:01, and HLA-DQB1*05:02) are associated with susceptibility to chronic hepatitis B (CHB), while decreased susceptibility to CHB was observed for HLA-DQB1*03:03 and HLA-DQB1*06:04. However, that study did not evaluate the association of HLA-DQB1 polymorphisms with spontaneous HBV clearance, and also did not include a large recent case–control study. Thus, it is unclear why some alleles are risk factors or protective factors for HBV infection. The present study aimed to examine the different HLA-DQB1 alleles, their associations with various cytokines (interferon-gamma [IFN-γ], interleukin-4 [IL-4], and interleukin-10 [IL-10]), and the various HLA-DQB1 mRNA expressions among HBV carriers and healthy controls. Furthermore, we performed a meta-analysis of the various alleles’ associations with HBV infection susceptibility and spontaneous HBV clearance.

## Methods

### Samples

The present study included samples from 433 healthy individuals who provided blood to the Deyang central blood station. A total of 256 patients with HBV infection were also enrolled during physical examinations at the Deyang People’s Hospital, Sichuan, China. Patients with HBV infection were identified has on serological results for HBsAg, antibodies to HBsAg, antibodies to Hepatitis Be, and antibodies to the HBV core antigen. Healthy controls (HCs) were seronegative for any HBV biomarkers. HBsAg/HBeAg/HBcAb/HBeAb/HBcAb-seropositive volunteers or patients were considered to be HBV carriers and all the patients were enrolled according to the related diagnostic criteria of 2010 “Chronic hepatitis B prevention and treatment guidelines” [17], HBV surface markers were used to determine the infected patients and the healthy controls. Since our research only aimed to explore the relationship between the DQB1 gene and HBV susceptibility, the relationship between DQB1 and the HBV infection process was not a main concern for us and therefore HCC and LC were not considered in this research. All blood samples were negative for the hepatitis C virus and human immunodeficiency virus. Asymptomatic carriers were defined as patients who were seropositive for HBsAg, had normal serum alanine aminotransferase and aspartate transaminase levels (< 35 U/L), and had no clear clinical symptoms without using antiretroviral drugs.

### HLA-DQB1 genotyping

The HLA-DQB1 genotyping was performed using peripheral blood samples that were collected into EDTA-coated tubes. High-quality genomic DNA were extracted from 400 µL of blood using a DNA extraction kit (Tiangen, Beijing, China), according to the manufacturer’s instructions. The DNA concentrations were 20–50 ng/µL, based on A260/280 values of 1.8–2.0. The genotyping was performed using PCR sequence-based typing and an ABI 3730 DNA Sequencer (Applied Biosystems, Foster City, CA). Amplification and sequencing primers were created using the sequences that were reported by van Dijk et al. [18]. The HLA-DQB1 alleles were identified based on the samples’ sequences using Utype software (Thermofisher, Waltham, USA).

### HLA-DQB1 mRNA measurements

The RNA were prepared from suspensions of freshly isolated peripheral blood mononuclear cells using a TRIzol method (Invitrogen, USA). The samples were treated using RNase-free DNase I (Qiagen, Germany) to eliminate contaminant genomic DNA, and then quantified using A260/280 measurements. Reverse transcription was used to obtain cDNA, based on 1 µg of total RNA and High Capacity cDNA Reverse Transcription Kits (Invitrogen), according to the manufacturer’s instructions. The mRNA expressions were quantified using SYBR green quantitative PCR and a CFX96 Touch PCR machine (Bio-Rad), based on the threshold cycle method. The forward primer sequence was TGGAGCACCCCAGCCT and the reverse primer sequence was ATSAGCCCCAGCACGAA. Each PCR tube included 12.5 µL of FastStart Universal SYBR Green Master (Roche, Switzerland), 200 nM of the primers, and 2.5 µL of cDNA in a total volume of 25 µL. The specificity of the reaction was confirmed using melt curve analysis for the dissociation step after the recommended Roche qPCR protocol, and all reactions were standardized using the expression of GAPDH.

### Meta-analysis

The effects of the HLA-DQB1 alleles on HBV infection susceptibility was evaluated using a meta-analysis. Relevant reports were identified using searches of the PubMed, EBSCO, Elsevier, and Web of Science databases up to September 2017. Reports in all languages were considered eligible for inclusion, and the search terms were set to ‘Hepatitis B’ and ‘HLA-DQB1’ without any other limits. The inclusion criteria were: (a) studies that analysed the genotype frequencies of HLA-DQB1 in HBV carriers and healthy HBV-uninfected controls, (b) studies that used a case–control design, (c) reports that provided the specific enrolment criteria, and (d) reports that included the number of cases, number of controls, and allele frequencies. All other studies were excluded.

### Statistical analysis

The Hardy–Weinberg equilibrium of the genotype distributions and the linkage disequilibrium of the single-nucleotide polymorphisms SNPs were examined using Arliquin software (version 3.5). Categorical and continuous variables were compared using the χ^2^ test and Student’s t test, respectively,multiple comparisons were adjusted by Bonferroni correction. The meta-analysis was performed using Review Manager Software (version 5.2), and the results were reported as ORs and 95% CIs. Heterogeneity between the studies was examined using the I^2^ test. The fixed-effect model was used if there was < 50% heterogeneity, otherwise the random-effect model was used. All tests were two-sided, and *P*-values of < 0.05 were considered statistically significant.

## Results

### Participant characteristics

The HBV carriers and healthy controls had similar values for age, sex, alanine transaminase, aspartate transaminase, albumin, and total bilirubin (Additional file 1: Table S1).

### Relationship between HLA-DQB1 and HBV infection susceptibility

The distributions of the HLA-DQB1 alleles in the HBV and healthy control groups were fit using the Hardy–Weinberg equilibrium. The results indicated that carriage of HLA-DQB1*06:03 protected against chronic HBV infection (odds ratio [OR] 0.009, 95% confidence interval [CI] 0.02–0.86) (Table 1).

**Table 1:** Distribution of the HLA-DQB1 alleles in hepatitis B virus infection and health control groups

HLA-DQB1*	HBV group		Healthy control		*P*	95% CI
	N = 256	%	N = 443	%		
02:01	49	9.57	87	9.82	0.926	0.97 (0.67–1.41)
02:02	–	–	2	0.23	–	–
03:01	97	18.95	168	18.96	1.00	1.00 (0.76–1.32)
03:02	31	6.05	49	5.53	0.55	1.15 (0.72–1.83)
03:03	86	16.80	153	17.27	0.88	0.97 (0.72–1.29)
04:01	24	4.69	43	4.85	1.00	0.96 (0.58–1.61)
04:02	4	0.78	10	1.13	0.59	0.69 (0.22–2.21)
05:01	19	3.71	36	4.06	0.78	0.91 (0.52–1.60)
05:02	78	15.23	113	12.75	0.20	1.23 (0.90–1.68)
05:03	30	5.86	34	3.84	0.085	1.56 (0.94–2.58)
06:01	58	11.33	99	11.17	0.93	1.02 (0.72–1.43)
06:02	22	4.30	47	31	0.44	0.80 (0.48–1.35)
06:03	1	0.20	15	1.69	*0.009* ^a^	0.11 (0.02–0.86)
06:04	–	–	10	1.13	–	–
06:05	11	2.15	13	1.47	0.39	1.47 (0.66–3.32)
06:09	2	0.39	5	0.56	1.00	0.69 (0.13–3.57)
06:10	–	–	2	0.23	–	–

*HBV* hepatitis B virus, *CI* confidence interval

^a^P < 0.01 (after Bonferroni correction)

Italic values indicate significance of* p* value (*p*≤0.05)

### Levels of IFN-γ, IL-4, and IL-10 in patients with HBV infection

**Fig. 1:**
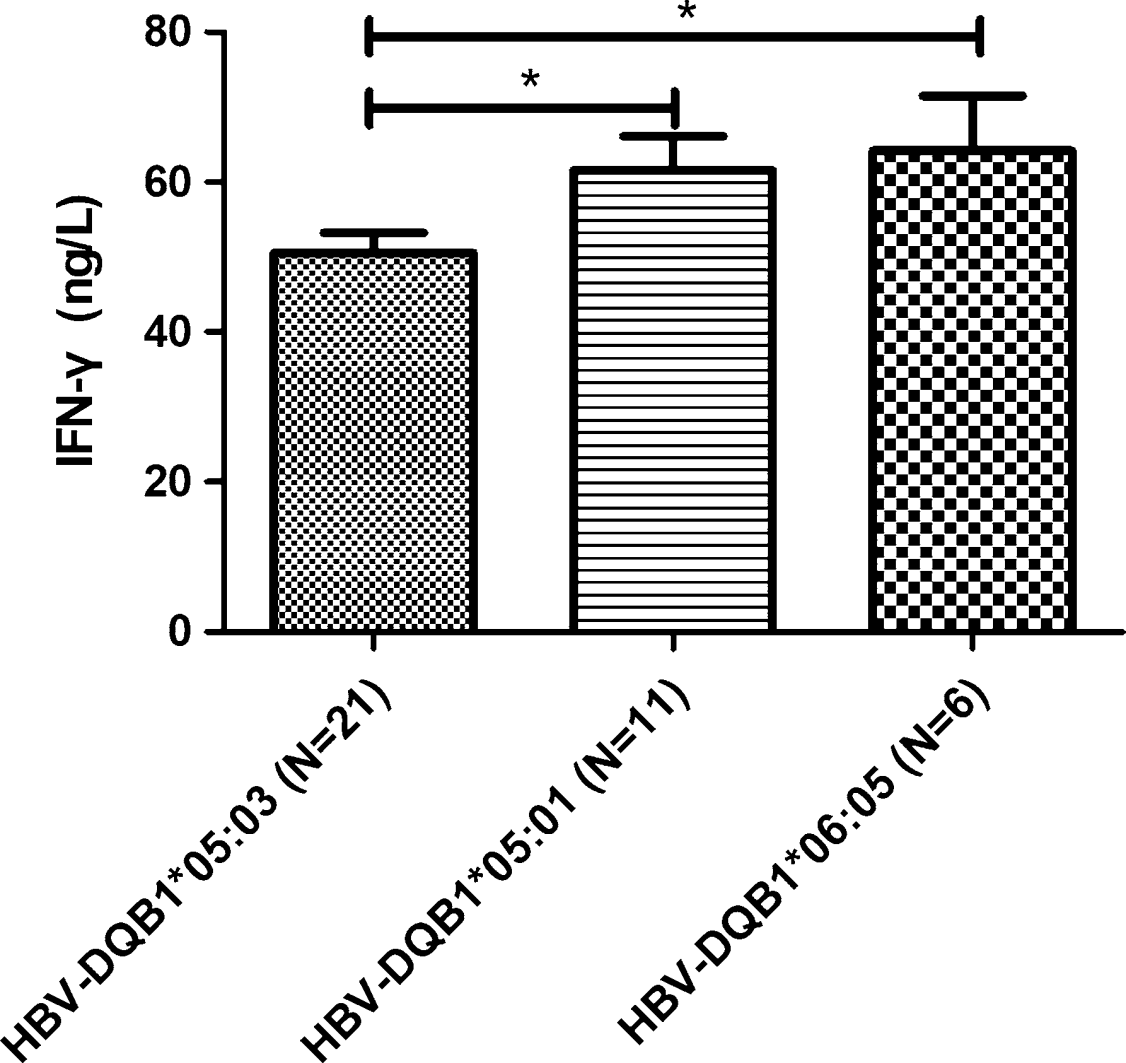
INF-γ levels according to different DQB1 alleles in the hepatitis B virus-infected group

**Fig. 2:**
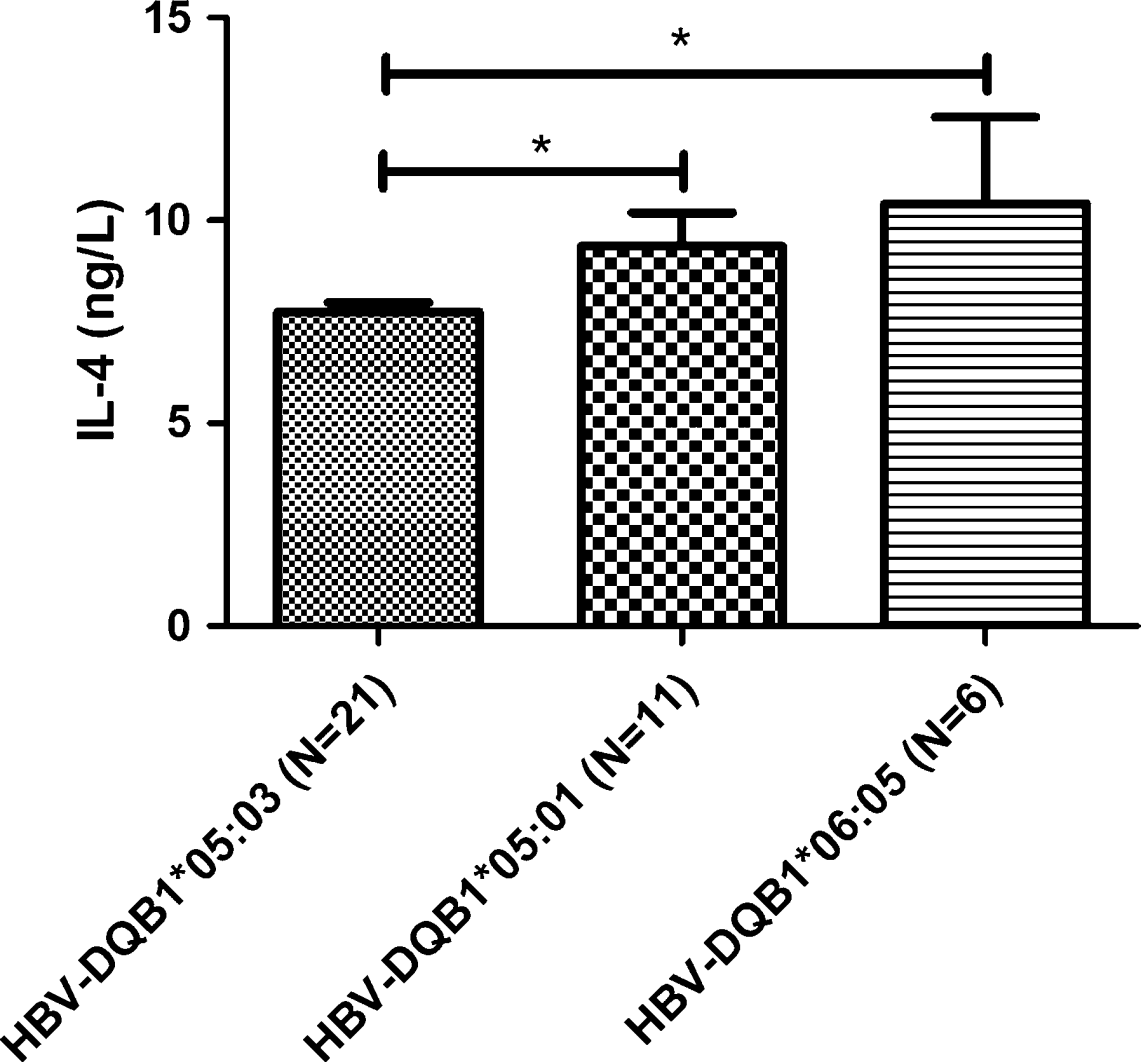
IL-4 levels according to different DQB1 alleles in the hepatitis B virus-infected group

Patients with HBV infection had significantly lower levels of IFN-γ, IL-4, and IL-10. In the HBV group, significantly higher levels of IFN-**γ** and IL-4 were associated with HLA-DQB1*05:01 and *06:05, compared to HLA-DQB1*05:03 (Figs. 1, 2). However, no significant differences in IL-10 levels were observed between the various HLA-DQB1 alleles (Additional file 1: Fig S1).

### HLA-DQB1 mRNA expression

**Fig. 3:**
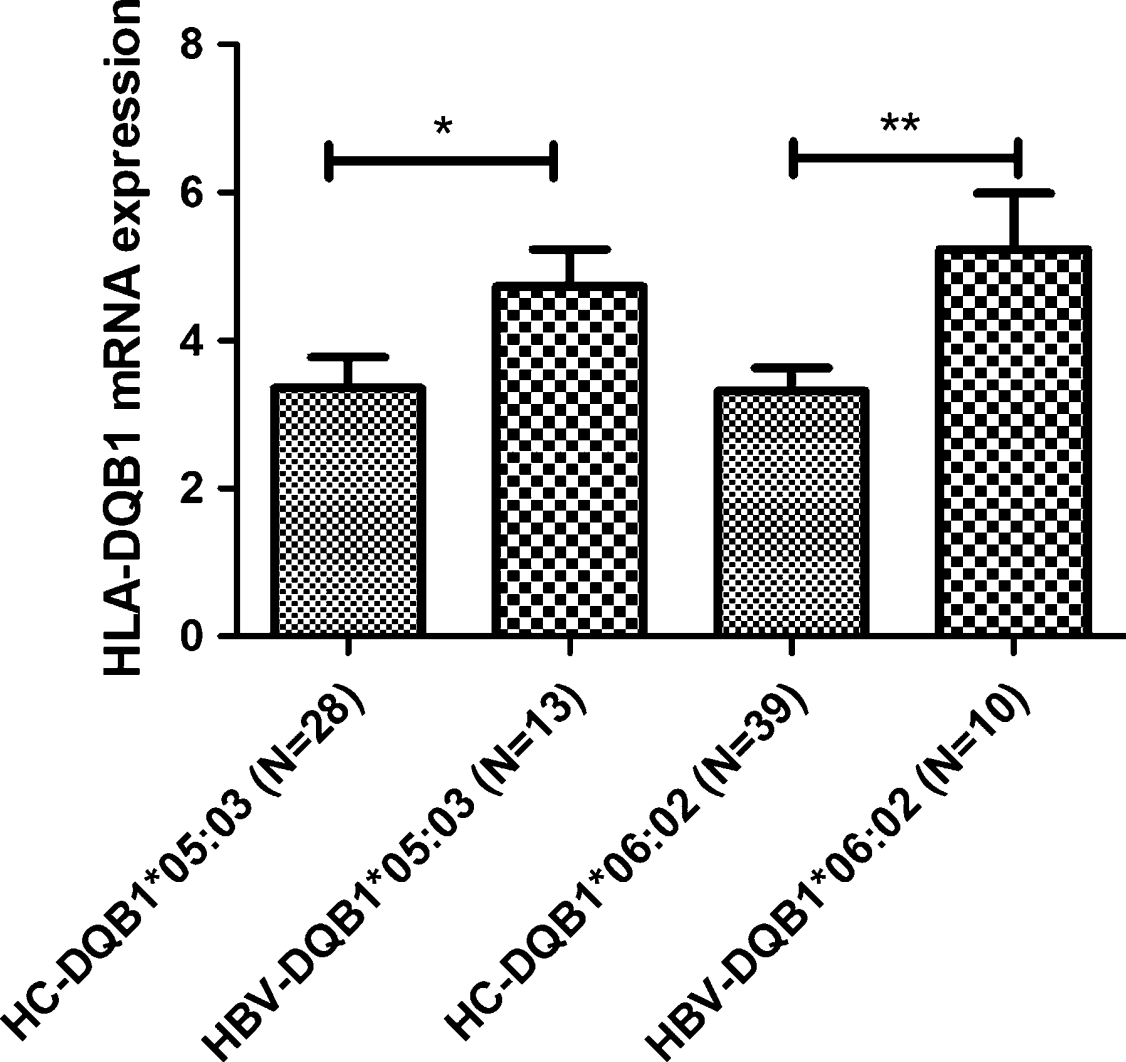
HLA-DQB1 mRNA expression levels in the hepatitis B virus-infected (HBV) and healthy control (HC) groups

Compared to the healthy control group, HLA-DQB1*05:03 and DQB1*06:02 in the HBV group were associated with significantly elevated HLA-DQB1 mRNA expression (Fig. 3).

### Meta-analysis of HLA-DQB1 alleles and HBV susceptibility

The meta-analysis evaluated 6 case–control studies regarding HLA-DQB1 and HBV infection susceptibility, which included 3459 cases of HBV infection and 7424 healthy controls (Table 2 and Additional file 1: Table S2). The risk factors for HBV infection susceptibility were HLA-DQB1*04:01 (OR 1.33, 95% CI 1.02–1.73, *P *= 0.03), HLA-DQB1*05:02 (OR 1.22, 95% CI 1.04–1.42, *P *= 0.01), HLA-DQB1*05:03 (OR 1.16, 95% CI 1.02–1.31, *P *= 0.02), and HLA-DQB1*06:01 (OR 1.32, 95% CI 1.02–1.70, *P *= 0.03). The factors that protected against HBV infection were HLA-DQB1*05:01 (OR 0.59, 95% CI 0.51–0.69, *P *< 0.00001), HLA-DQB1*06:03 (OR 0.28, 95% CI 0.08–1.00, *P *= 0.05), and HLA-DQB1*06:04 (OR 0.31, 95% CI 0.20–0.49, *P *< 0.00001) (Table 3). The forest plots are shown in Additional file 1: Figures S2–S8.

**Table 2:** Characteristics of the studies regarding hepatitis B virus infection susceptibility

First author	Year	Ethnicity	Total		Genotyping method	Refs
			HBV carriers (n)	Healthy controls (n)		
Nishida	2016	Japanese	805	2278	Array assay	[21]
Mbarek	2011	Japanese	2209	4404	Array assay	[19]
Jiang	2003	Chinese Han	82	106	PCR-SSP	[28]
Park	2003	Korean	89	100	RFLP and PCR-SSCP	[22]
Chen	1996	Caucasian	58	106	PCR-SSO	[23]

*HBV* hepatitis B virus, *Refs* references, *PCR* polymerase chain reaction, *SSP* single specific primer, *RFLP* restriction fragment length polymorphism, *SSCP* single-strand conformational polymorphism, *SSO* sequence-specific oligonucleotide

**Table 3:** Overall results regarding HLA-DQB1 polymorphisms in hepatitis B virus-susceptible patients

HLA-DQB1*	N	Heterogeneity			Overall relationship	
		I^2^ (%)	*P*	Model	OR (95% CI)	*P*
02:01	5	0	0.43	F	0.96 (0.74–0.23)	0.74
03:01	6	63	0.02	R	1.06 (0.87–1.28)	0.58
03:02	6	66	0.03	R	0.83 (0.64–1.07)	0.16
03:03	6	78	0.0004	R	1.20 (0.95–1.52)	0.13
*04:01*	5	82	0.0001	R	1.33 (1.02–1.73)	*0.03*
04:02	6	41	0.13	F	0.89 (0.78–1.02)	0.09
*05:01*	6	46	0.10	F	0.59 (0.51–0.69)	*< 0.00001*
*05:02*	6	0	0.55	F	1.22 (1.04–1.42)	*0.01*
*05:03*	6	0	0.76	F	1.16 (1.02–1.31)	*0.02*
*06:01*	5	83	< 0.00001	R	1.32 (1.02–1.70)	*0.03*
06:02	6	91	< 0.00001	R	1.13 (0.66–1.96)	0.65
*06:03*	5	67	0.02	R	0.28 (0.08–1.00)	*0.05*
*06:04*	5	64	0.03	R	0.31 (0.20–0.49)	*< 0.00001*

*OR* odds ratio, *CI* confidence interval, *F* fix effects model, *R* random effects model

Italic values indicate significance of* p* value (*p*≤0.05)

### Meta-analysis of HLA-DQB1 alleles and spontaneous HBV clearance

The meta-analysis evaluated 7 case–control studies regarding HLA-DQB1 and spontaneous HBV clearance, which included 602 cases with spontaneous HBV clearance and 954 cases with chronic HBV infection (Table 4 and Additional file 1: Table S3). Spontaneous HBV clearance was significantly associated with HLA- DQB1*06:04 (OR 2.00, 95% CI 1.07–3.74, *P *= 0.03). Chronic HBV infection was significantly associated with HLA-DQB1*02:01 (OR 0.77, 95% CI 0.60–0.98, *P *= 0.04) and HLA-DQB1*05:02 (OR 0.44, 95% CI 0.27–0.70, *P *= 0.0006) (Table 5). The forest plots are shown in Additional file 1: Figures S9–S11.

**Table 4:** Meta-analysis of HLA-DQB1 polymorphisms and hepatitis B virus clearance

First author	Year	Ethnicity	Total		Genotyping method	Refs
			Spontaneous clearance	Chronic HBV infections		
Zhang	2015	Chinese (Uygur)	80	110	PCR-SSP	[18]
Cho	2008	Koreans	80	384	PCR-SBT	[24]
Liu	2007	Chinese (Han)	100	168	PCR-SSP	[25]
Zhu	2007	Chinese (Han)	133	151	PCR-SSP	[20]
Jiang	2003	Chinese (Han)	30	52	PCR-SSP	[28]
Thio	1999	African American	60	31	PCR-SSP	[29]
Chen	1996	Caucasian	119	58	PCR-SSO	[23]

*HBV* hepatitis B virus, *Refs* references, *PCR* polymerase chain reaction, *SSP* single specific primer, *SBT* sequenced-based typing, *SSO* sequence-specific oligonucleotide

**Table 5:** Meta-analysis of HLA-DQB1 alleles associated with hepatitis B virus spontaneous clearance

HLA-DQB1*	N	Heterogeneity			Overall relationship	
		I^2^ (%)	*P*	Model	OR (95% CI)	*P*
*02:01*	6	47	0.09	F	0.77 (0.60–0.98)	*0.04*
03:01	5	82	0.0002	R	0.52 (0.25–1.06)	0.07
03:02	5	56	0.09	R	1.34 (0.85–2.11)	0.21
03:03	5	0	0.86	F	1.18 (0.93–1.51)	0.17
04:01	5	0	0.69	F	1.16 (0.78–1.73)	0.46
04:02	4	0	0.57	F	1.11 (0.59–2.10)	0.75
05:01	6	0	0.65	F	1.04 (0.73–1.49)	0.82
*05:02*	6	9	0.36	F	0.44 (0.27–0.70)	*0.0006*
05:03	4	0	0.95	F	0.81 (0.50–1.33)	0.41
06:01	4	40	0.17	F	0.77 (0.56–1.06)	0.11
06:02	6	0	0.97	F	1.25 (0.97–1.61)	0.09
06:03	3	0	0.45	F	1.20 (0.47–3.08)	0.70
*06:04*	5	0	0.98	F	2.00 (1.07–3.74)	*0.03*

*OR* odds ratio, *CI* confidence interval, *F* fix effects model, *R* random effects model

Italic values indicate significance of* p* value (*p*≤0.05)

## Discussion

The associations between HLA expression and HBV infection or clearance have been intensively assessed across various populations^[19−21]^. For example, Nishda et al. [22] evaluated 1033 Japanese patients with HBV infection and 942 healthy controls, and found that HLA-DQB1*06:01 had the strongest association with CHB susceptibility. Mbarek et al. [20] also examined three independent Japanese cohorts (2209 CHB cases and 4440 controls), and found that HLA-DQA1∗01:02-DQB1∗06:04 and HLA-DQA1∗01:01-DQB1∗05:01 protected against CHB, while HLA-DQA1∗01:02-DQB1∗03:03 and HLA-DQA1∗03:01-DQB1∗06:01 were risk factors for CHB. Furthermore, Zhang et al. [19]. found that HLA-DQB1*03:01 was closely related to CHB susceptibility, while Park et al. [23] reported that HLA-DQB1*04:02 and HLA-DQB1*06:04 protected against CHB. Chen et al. [24] confirmed that HLA-DQ1*06:04 was associated with susceptibility to HBV infection among Caucasian individuals. However, in our Sichuan population, we found that HLA-DQB1*06:03 independently protected against HBV infection. Given the varying results in the different studies and patient populations, we performed a meta-analysis of 6 reports that included Chinese, Japanese, Korean, and Caucasian patients. Among the 3459 cases of HBV infection and 7424 healthy controls, susceptibility to HBV infection was associated with HLA-DQB1*04:01, HLA-DQB1*05:02, HLA-DQB1*05:03, and HLA-DQB1*06:01. However, HLA-DQB1*05:01, HLA-DQB1*06:03, and HLA-DQB1*06:04 protected against HBV infection.

The associations of HLA expression with spontaneous HBV clearance has also been investigated in several studies. For example, Zhou et al. [19] found that HLA-DQB1*02:01 protected against HBV infection, while HLA-DQB1*03:01 was associated with chronic HBV infection in Xinjiang. Cho et al. [25] have also found that HLA-DQB1*06:09 was unexpectedly and strongly associated with HBV clearance. Furthermore, Liu et al. [26] demonstrated that HLA-DQB1*02:01 and HLA-DQB1*06:01 conferred susceptibility to chronic HBV infection. Zhu et al. [21] have also reported that HLA-DQB1*05:02 was independently associated with the outcomes of HBV infection. Thus, we performed a meta-analysis of the 7 reports that included Chinese, Korean, African American, and Caucasian patients. This analysis revealed that spontaneous HBV clearance was significantly associated with HLA- DQB1*06:04, while chronic HBV infection was significantly associated with HLA-DQB1*02:01 and HLA-DQB1*05:02. An easy conclusion is that DQB1*05:02 was not only associated with susceptible of HBV infection, but also with persistent chronic HBV infection. The DQB1*06:04, on the other hand, was related to HBV protection and HBV spontaneous clearance. We also found that some risk alleles or protect alleles varies between different researches. This heterogeneity was also shown in the meta-analysis: the heterogeneity (I^2^ > 50%) between DQB1 alleles and HBV susceptibility were obviously for DQB1*04:01, 06:01, 06:03 and 06:04. As host-virus immune interaction might be influenced by viral genotype. Doganay et al. reported [27] that DQB1 05:01 was associated with chronic active disease where all patients were exclusively genotype D. As we know, genotype B and C were dominantly prevalent in Chinese HBV infections while the HBV genotype in European and American infections were mainly A and D. Therefore, the difference in the HBV virus genotype in different regions may also one important factor for HBV susceptibility and could account for the heterogeneity of the DQB1 gene polymorphism and HBV infection outcomes.

APCs express HLA-II molecules (HLA-DR, HLA-DQ, and HLA-DP) to activate CD4+ helper T-cells, which play a crucial role in the immune response to HBV infection. The present study revealed that HBV infection was associated with significantly lower levels of INF-γ, IL-4, and IL-10, while previous studies have not examined this issue. Furthermore, we observed that HLA-DQB1*05:03 in the HBV group was associated with significantly lower IFN-γ and IL-4 levels, and the meta-analysis revealed that HLA-DQB1*05:03 was associated with HBV infection. Thus, the lower levels of IFN-γ and IL-4 may reflect a reduced immune response in cases with this allele, which could increase susceptibility to HBV.

O’Brien et al. [28] have reported that the rs3077TT genotype was associated with higher HLA-DPB1 mRNA expression in the liver, which was also associated with a lower risk of chronic HBV infection. In addition, Thomas et al. [9] demonstrated that the rs9277534GG genotype confers susceptibility to chronic HBV infection, with significantly decreased levels of HLA-DP transcripts and surface protein in HBV donors. In contrast, the present study revealed that plasma expression of HLA-DQB1 mRNA was elevated in cases with HLA-DQB1*05:03, which was associated with HBV infection susceptibility. A previous study [29] also detected elevated HLA-DQB1 mRNA expression in cases with HLA-DQB1*06:02, which was associated with narcolepsy, We also found that the risk allele DQB1*05:03 have significantly lower IFN-γ and IL-4 levels compared than protect allele DQB1*05:01, at the same time, the immune negative regulatory factor IL-10 have no difference between risk alleles and protect alleles in HBV group. From these results, we could postulate that some risk DQB1 alleles may increase DQB1 mRNA expression, resulting in decreased immunity related cytokines. Thus, additional studies and large population studies are needed to understand the relevance of HLA-DQB1 expression, especially in the context of APCs and different HBV genotype-specific antigens.

## Conclusion

HBV infection susceptibility was associated with HLA-DQB1*04:01, HLA-DQB1*05:02, HLA-DQB1*05:03, and HLA-DQB1*06:01, while protection against HBV infection was associated with HLA-DQB1*05:01, HLA-DQB1*06:03, and HLA-DQB1*06:04. Spontaneous HBV clearance was associated with HLA-DQB1*02:01 and HLA-DQB1*05:02, while chronic HBV infection was associated with HLA-DQB1*06:04. In cases of HBV infection, HLA-DQB1*05:03 was associated with low levels of IFN-γ andIL-4 secretion, and with increased HLA-DQB1 mRNA expression.

## Additional file

**Additional file 1: Table S1** The characteristic of the participates. **Table S2**. Distribution of HLA-DQB1 alleles in different studies. **Table S3**. Distribution of the HLA-DQB1 polymorphism in HBV spontaneous clearance. **Figure S1**. IL-10 with different DQB1 alleles in HBV group. **Figure S2**. Meta-analysis of correlation of the HLA-DQB1*04:01 allele polymorphism in HBV susceptibility. **Figure S3**. Meta-analysis of correlation of the HLA-DQB1*05:01 allele polymorphism in HBV susceptibility. **Figure S4**. Meta-analysis of correlation of the HLA-DQB1*05:02 allele polymorphism in HBV susceptibility. **Figure S5**. Meta-analysis of correlation of the HLA-DQB1*05:03 allele polymorphism in HBV susceptibility. **Figure S6**. Meta-analysis of correlation of the HLA-DQB1*06:01 allele polymorphism in HBV susceptibility. **Figure S7**. Meta-analysis of correlation of the HLA-DQB1*06:03 allele polymorphism in HBV susceptibility. **Figure S8**. Meta-analysis of correlation of the HLA-DQB1*06:04 allele polymorphism in HBV susceptibility. **Figure S9**. Meta-analysis of correlation of the HLA-DQB1*02:01 allele polymorphism in HBV spontaneous clearance. **Figure S10**. Meta-analysis of correlation of the HLA-DQB1*05:02 allele polymorphism in HBV spontaneous clearance. **Figure S11.** Meta-analysis of correlation of the HLA-DQB1*06:04 allele polymorphism in HBV spontaneous clearance.

## Abbreviations

HBV: hepatitis B virus
IFN-γ: interferon-gamma
IL-4: interleukin-4
IL-10: interleukin-10
HBsAg: hepatitis B surface antigen
HLA: human leukocyte antigens
APCs: antigen-presenting cells
