# Supplementary material for: The roles of HLA-DQB1 gene polymorphisms in hepatitis B virus infection
Source: J Transl Med. 2018 Dec 18;16:362. doi: 10.1186/s12967-018-1716-z (PMC6299522; doi:10.1186/s12967-018-1716-z)
Supplement: Supplementary file 1 — Additional file 1: Table S1 The characteristic of the participates. Table S2. Distribution of HLA-DQB1 alleles in different studies. Table S3. Distribution of the HLA-DQB1 polymorphism in HBV spontaneous clearance. Figure S1. IL-10 with different DQB1 alleles in HBV group. Figure S2. Meta-analysis of correlation of the HLA-DQB1*04:01 allele polymorphism in HBV susceptibility. Figure S3. Meta-analysis of correlation of the HLA-DQB1*05:01 allele polymorphism in HBV susceptibility. Figure S4. Meta-analysis of correlation of the HLA-DQB1*05:02 allele polymorphism in HBV susceptibility. Figure S5. Meta-analysis of correlation of the HLA-DQB1*05:03 allele polymorphism in HBV susceptibility. Figure S6. Meta-analysis of correlation of the HLA-DQB1*06:01 allele polymorphism in HBV susceptibility. Figure S7. Meta-analysis of correlation of the HLA-DQB1*06:03 allele polymorphism in HBV susceptibility. Figure S8. Meta-analysis of correlation of the HLA-DQB1*06:04 allele polymorphism in HBV susceptibility. Figure S9. Meta-analysis of correlation of the HLA-DQB1*02:01 allele polymorphism in HBV spontaneous clearance. Figure S10. Meta-analysis of correlation of the HLA-DQB1*05:02 allele polymorphism in HBV spontaneous clearance. Figure S11. Meta-analysis of correlation of the HLA-DQB1*06:04 allele polymorphism in HBV spontaneous clearance. [file 12967_2018_1716_MOESM1_ESM.doc]

**Additional figures and tables**

**The roles of HLA-DQB1 gene polymorphisms in hepatitis B virus infection**

**Running title:** HLA-DQB1 gene polymorphisms and HBV infection

Guojin Ou^1,2,3,4^, Haixia Xu^3,4^, Hao Yu^3,4^, Xiao Liu^5^, Liu Yang^6^, Xin Ji^3,4^, Jue Wang^3,4^*, Zhong Liu^3,4^*

1. Department of laboratory medicine, West China second university hospital, Chengdu, Sichuan, China.

2. Key Laboratory of Birth Defects and Related Diseases of Women and Children (Sichuan University), Ministry of Education.

3. Clinical Transfusion Research Center, Institute of Blood Transfusion, CAMS & PUMC, Chengdu, Sichuan, China.

4. Key laboratory of transfusion adverse reactions, CAMS, Chengdu, Sichuan, China.

5. Peoples Hospital of Deyang City, Sichuan, China.

6. Tianfu new district people’s hospital, Chengdu, Sichuan, China.

Guojin Ou: jiaozhu327@163.com

Haixia Xu: 530704473@qq.com

Hao Yu: 943287441@qq.com

Xiao Liu: 354211645@qq.com

Liu Yang: 147677670@qq.com

Xin Ji: 380394746@qq.com

***Correspondence**

Zhong Liu, Jue Wang

Clinical Blood Transfusion Research Center, Institute of Blood Transfusion, CAMS & PUMC, Chengdu, Sichuan 610052, China.

Address: Zhong Liu, No. 26 Hua-Cai Road, Chenghua District, Chengdu, Sichuan Provence, 610052, China. E-mail: liuz@ibt.pumc.edu.cn, wjue007@qq.com

**Conflicts of interest.** The authors confirm that they have no conflicts of interest.

Table S1 The characteristic of the participates

|  | Age (years) | Male | ALT(IU/L) | AST(IU/L) | ALB(g/dl) | TB(mg/dl) |
| --- | --- | --- | --- | --- | --- | --- |
| HBV carriers(N=256) | 52.2±8 | 141(55.1%) | 23.5±4.3 | 23±2.8 | 4.3±1.0 | 1.4±0.6 |
| HC group(N=443) | 55.4±13 | 258(58.2%) | 22.6±3.3 | 24±4.4 | 3.6±0.9 | 1.2±0.7 |


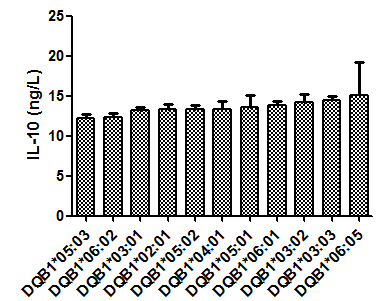


Figure S1 IL-10 with different DQB1 alleles in HBV group.

Table S2 Distribution of HLA-DQB1 alleles in different studies.

| First author (year) | HLA-DQB1 alleles | | | | | | | | | | | | | | | Refs |
| --- | --- | --- | --- | --- | --- | --- | --- | --- | --- | --- | --- | --- | --- | --- | --- | --- |
|  | 02:01 | 02:02 | 03:01 | 03:02 | 03:03 | 04:01 | 04:02 | 05:01 | 05:02 | 05:03 | 06:01 | 06:02 | 06:03 | 06:04 | 06:09 |  |
| Nishida N (2016) | |  |  |  |  |  |  |  |  |  |  |  |  |  |  | [1] |
| Case, n | 3 | NA | 153 | 85 | 325 | 213 | 47 | 52 | 38 | 63 | 479 | 112 | 1 | 37 | 2 |  |
| Control, n | 17 | NA | 502 | 394 | 737 | 608 | 179 | 310 | 97 | 157 | 883 | 337 | 23 | 291 | 21 |  |
| Mbarek H(2011) | |  |  |  |  |  |  |  |  |  |  |  |  |  |  | [2] |
| Case, n | NA | 30 | 449 | 281 | 924 | 508 | 260 | 133 | 136 | 298 | 1066 | 239 | NA | 95 | NA |  |
| Control, n | NA | 107 | 875 | 624 | 1219 | 1319 | 552 | 509 | 208 | 531 | 1793 | 194 | NA | 803 | NA |  |
| Jiang YG (2003) | |  |  |  |  |  |  |  |  |  |  |  |  |  |  | [3] |
| Case, n | 16 | NA | 53 | 9 | 25 | 8 | 2 | 5 | 10 | 3 | 14 | 7 | 3 | 4 | NA |  |
| Control, n | 23 | NA | 40 | 14 | 35 | 11 | 2 | 9 | 20 | 6 | 20 | 12 | 5 | 7 | NA |  |
| Chen DF (1996) | |  |  |  |  |  |  |  |  |  |  |  |  |  |  | [4] |
| Case, n | 23 | NA | 19 | 10 | 6 | NA | 4 | 8 | 3 | 6 | 0 | 14 | 7 | 1 | NA |  |
| Control, n | 49 | NA | 30 | 28 | 9 | NA | 1 | 21 | 6 | 7 | 1 | 19 | 14 | 13 | NA |  |
| Park MH(2003) | |  |  |  |  |  |  |  |  |  |  |  |  |  |  | [5] |
| Case, n | 19 | NA | 30 | 21 | 17 | 14 | 3 | 7 | 5 | 13 | 17 | 14 | 1 | 2 | 4 |  |
| Control, n | 13 | NA | 26 | 18 | 24 | 9 | 11 | 17 | 3 | 11 | 12 | 18 | 5 | 14 | 5 |  |


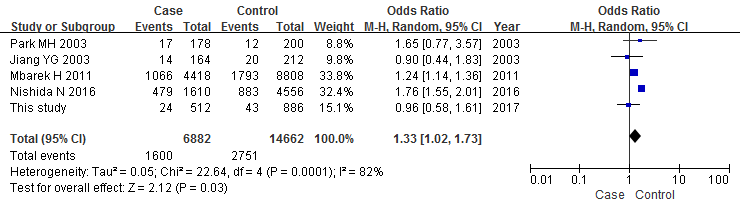


Figure S2 Meta-analysis of correlation of the HLA-DQB1*04:01 allele polymorphism in HBV susceptibility


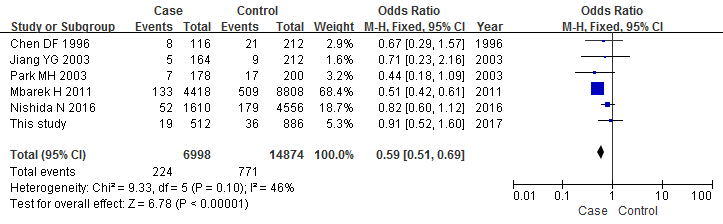


Figure S3 Meta-analysis of correlation of the HLA-DQB1*05:01 allele polymorphism in HBV susceptibility


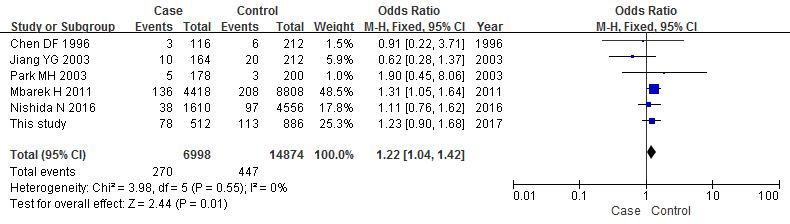


Figure S4 Meta-analysis of correlation of the HLA-DQB1*05:02 allele polymorphism in HBV susceptibility


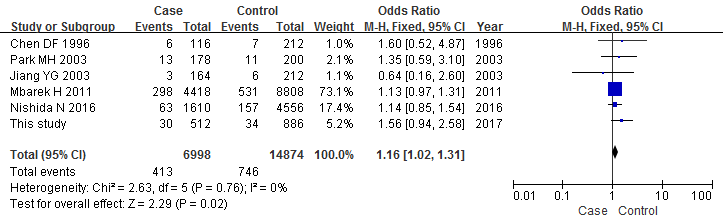


Figure S5 Meta-analysis of correlation of the HLA-DQB1*05:03 allele polymorphism in HBV susceptibility
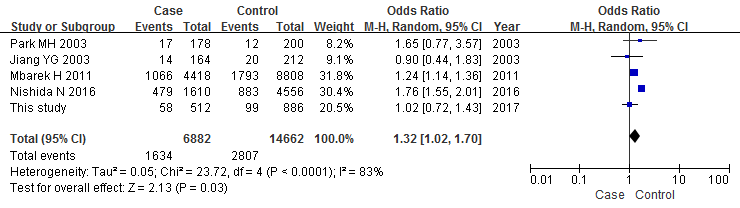


Figure S6 Meta-analysis of correlation of the HLA-DQB1*06:01 allele polymorphism in HBV susceptibility
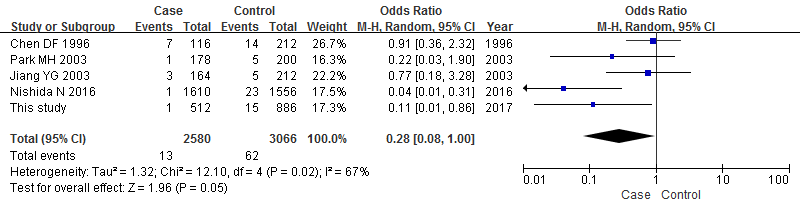


Figure S7 Meta analysis of correlation of the HLA-DQB1*06:03 allele polymorphism in HBV susceptibility


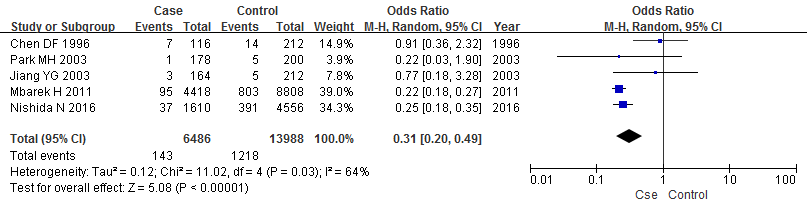


Figure S8 Meta analysis of correlation of the HLA-DQB1*06:04 allele polymorphism in HBV susceptibility

Table S3 Distribution of the HLA-DQB1 polymorphism in HBV spontaneous clearance

| First author (year) | HLA-DQB1 alleles | | | | | | | | | | | | | | | | Refs |
| --- | --- | --- | --- | --- | --- | --- | --- | --- | --- | --- | --- | --- | --- | --- | --- | --- | --- |
|  | 0201 | 202 | 301 | 302 | 303 | 401 | 402 | 501 | 502 | 503 | 601 | 602 | 603 | 604 | 607 | 609 |  |
| Zhang Y (2015) | | | | | | | | | | | | | | | | | [6] |
| SC, n | 30 | NA | 35 | NA | 39 | 6 | NA | 8 | 6 | NA | NA | 15 | NA | 1 | NA | NA |  |
| CHB, n | 39 | NA | 59 | NA | 43 | 7 | NA | 10 | 13 | NA | NA | 19 | NA | 1 | NA | NA |  |
| Cho SW (2008) | | | | | | | | | | | | | | | | | [7] |
| SC, n | 2 | 7 | 17 | 20 | 21 | 11 | 6 | 9 | 1 | 8 | 20 | 12 | NA | 6 | NA | 8 |  |
| CHB, n | 20 | 65 | 126 | 49 | 96 | 56 | 22 | 50 | 27 | 41 | 99 | 54 | NA | 14 | NA | 6 |  |
| Liu C (2007) | | | | | | | | | | | | | | | | | [8] |
| SC, n | 17 | NA | 5 | 8 | 12 | 10 | 5 | 2 | 3 | 8 | 15 | 52 | 3 | 7 | 3 | NA |  |
| CHB, n | 63 | NA | 10 | 14 | 14 | 9 | 5 | 7 | 9 | 16 | 45 | 67 | 7 | 7 | 3 | NA |  |
| Zhu XL(2007) | | | | | | | | | | | | | | | | | [9] |
| SC, n | 38 | NA | NA | 20 | 61 | 10 | NA | 18 | 3 | NA | 15 | 25 | NA | NA | NA | NA |  |
| CHB, n | 51 | NA | NA | 12 | 61 | 8 | NA | 15 | 18 | NA | 26 | 22 | NA | NA | NA | NA |  |
| Jiang YG (2003) | | | | | | | | | | | | | | | | | [3] |
| SC, n | 6 | NA | NA | 16 | 10 | 5 | 1 | 3 | 7 | 2 | 7 | 3 | 1 | 2 | NA | NA |  |
| CHB, n | 10 | NA | NA | 37 | 15 | 11 | 2 | 9 | 20 | 6 | 7 | 4 | 2 | 2 | NA | NA |  |
| Thio CL (1999) | |  |  |  |  |  |  |  |  |  |  |  |  |  |  |  | [10] |
| SC, n | NA | NA | 12 | NA | NA | NA | NA | NA | NA | NA | NA | NA | NA | NA | NA | NA |  |
| CHB, n | NA | NA | 32 | NA | NA | NA | NA | NA | NA | NA | NA | NA | NA | NA | NA | NA |  |
| Chen DF (1996) | | | | | | | | | | | | | | | | | [4] |
| SC, n | 46 | NA | 39 | 28 | 8 | NA | 4 | 23 | 7 | 9 | 2 | 31 | 10 | 7 | NA | NA |  |
| CHB, n | 23 | NA | 19 | 10 | 6 | NA | 4 | 8 | 3 | 6 | 0 | 14 | 7 | 1 | NA | NA |  |


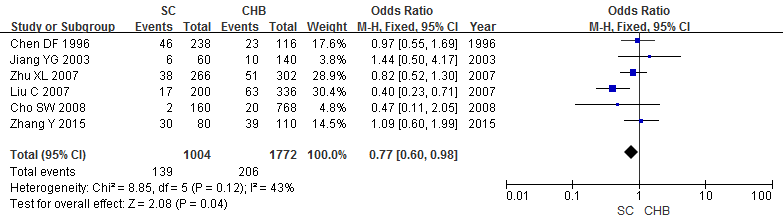


Figure S9 Meta-analysis of correlation of the HLA-DQB1*02:01 allele polymorphism in HBV spontaneous clearance


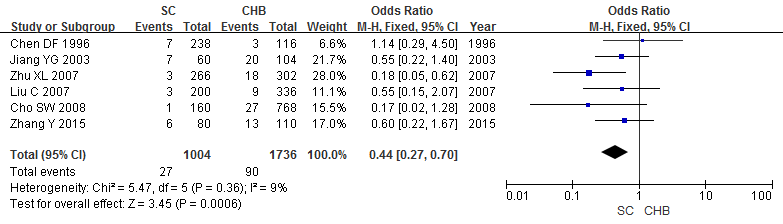


Figure S10 Meta-analysis of correlation of the HLA-DQB1*05:02 allele polymorphism in HBV spontaneous clearance


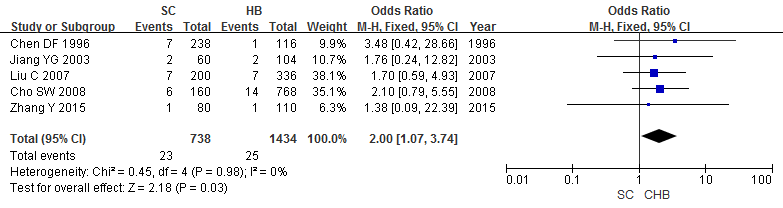


Figure S11 Meta-analysis of correlation of the HLA-DQB1*06:04 allele polymorphism in HBV spontaneous clearance

1. Nishida N, Ohashi J, Khor SS, Sugiyama M, Tsuchiura T, Sawai H, Hino K, Honda M, Kaneko S, Yatsuhashi H, et al: **Understanding of HLA-conferred susceptibility to chronic hepatitis B infection requires HLA genotyping-based association analysis.** *Sci Rep* 2016, **6:**24767.

2. Mbarek H, Ochi H, Urabe Y, Kumar V, Kubo M, Hosono N, Takahashi A, Kamatani Y, Miki D, Abe H, et al: **A genome-wide association study of chronic hepatitis B identified novel risk locus in a Japanese population.** *Hum Mol Genet* 2011, **20:**3884-3892.

3. Jiang YG, Wang YM, Liu TH, Liu J: **Association between HLA class II gene and susceptibility or resistance to chronic hepatitis B.** *World J Gastroenterol* 2003, **9:**2221-2225.

4. Chen DF, Kliem V, Endres W, Brunkhorst R, Tillmann HL, Koch KM, Manns MP, Stangel W: **Relationship between human leukocyte antigen determinants and courses of hepatitis B virus infection in Caucasian patients with end-stage renal disease.** *Scand J Gastroenterol* 1996, **31:**1211-1215.

5. Park MH, Song EY, Ahn C, Oh KH, Yang J, Kang SJ, Lee HS: **Two subtypes of hepatitis B virus-associated glomerulonephritis are associated with different HLA-DR2 alleles in Koreans.** *Tissue Antigens* 2003, **62:**505-511.

6. Zhang Y, Zhao F, Lan L, Qin Z, Jun L: **Correlation of HLA-DQB1 gene polymorphism of Xinjiang Uygur with outcome of HBV infection.** *Int J Clin Exp Med* 2015, **8:**6067-6072.

7. Cho SW, Cheong JY, Ju YS, Oh DH, Suh YJ, Lee KW: **Human leukocyte antigen class II association with spontaneous recovery from hepatitis B virus infection in Koreans: analysis at the haplotype level.** *J Korean Med Sci* 2008, **23:**838-844.

8. Liu C, Cheng B: **Association of polymorphisms of human leucocyte antigen-DQA1 and DQB1 alleles with chronic hepatitis B virus infection, liver cirrhosis and hepatocellular carcinoma in Chinese.** *Int J Immunogenet* 2007, **34:**373-378.

9. Zhu XL, Du T, Li JH, Lu LP, Guo XH, Gao JR, Gou CY, Li Z, Liu Y, Li H: **Association of HLA-DQB1 gene polymorphisms with outcomes of HBV infection in Chinese Han population.** *Swiss Med Wkly* 2007, **137:**114-120.

10. Thio CL, Carrington M, Marti D, O'Brien SJ, Vlahov D, Nelson KE, Astemborski J, Thomas DL: **Class II HLA alleles and hepatitis B virus persistence in African Americans.** *J Infect Dis* 1999, **179:**1004-1006.
